# Primary care-based educational interventions to decrease risk factors for metabolic syndrome for adults with major psychotic and/or affective disorders: a systematic review

**DOI:** 10.1186/2046-4053-2-116

**Published:** 2013-12-27

**Authors:** Cynthia Nover, Sarah S Jackson

**Affiliations:** 1 College of Social & Behavioral Sciences and Social Work, Eastern Washington University, 208 Senior Hall, Cheney, WA 99004, USA; 2 Department of Epidemiology/Biostatistics, George Washington University, School of Public Health, 2100 W. Pennsylvania Ave., 8th Floor, Washington, DC 20037, USA

**Keywords:** Metabolic syndrome, Serious mental illness, Primary care, Weight loss, Schizophrenia, Schizoaffective, Bipolar, Major depressive disorder, Physical health intervention, Educational intervention, Behavioral intervention

## Abstract

**Background:**

Individuals with major psychotic and/or affective disorders are at increased risk for developing metabolic syndrome due to lifestyle- and treatment-related factors. Numerous pharmacological and non-pharmacological interventions have been tested in inpatient and outpatient mental health settings to decrease these risk factors. This review focuses on primary care-based non-pharmacological (educational or behavioral) interventions to decrease metabolic syndrome risk factors in adults with major psychotic and/or affective disorders.

**Methods:**

The authors conducted database searches of PsychINFO, MEDLINE and the Cochrane Database of Systematic Reviews, as well as manual searches and gray literature searches to identify included studies.

**Results:**

The authors were unable to identify any studies meeting *a priori* inclusion criteria because there were no primary care-based studies.

**Conclusions:**

This review was unable to demonstrate effectiveness of educational interventions in primary care. Interventions to decrease metabolic syndrome risk have been demonstrated to be effective in mental health and other outpatient settings. The prevalence of mental illness in primary care settings warrants similar interventions to improve health outcomes for this population.

## Background

### Comorbidity of mental illness and chronic physical illness

Individuals with major psychotic and/or affective disorders (for example, schizophrenia, bipolar disorder or major depressive disorder) experience higher rates of comorbid physical health problems compared with the general population. Cardiovascular risk and metabolic risk are increased in individuals with schizophrenia [1-3] and depression [4,5]. Bipolar disorder has also been shown to be associated with metabolic syndrome [6,7]. Risk factors for cardiovascular disease and metabolic syndrome include: high blood pressure, large waist circumference, high triglyceride levels, low HDL cholesterol level, and high fasting blood sugar levels.

Causes of comorbidity in this population are thought to include psychiatric medication and lifestyle factors, such as diet and tobacco consumption. Atypical antipsychotic medication (AAP), commonly prescribed for patients with bipolar disorder or schizophrenia, increases risk for metabolic syndrome [8-10]. Individuals with major psychotic disorders, especially schizophrenic disorders, also consume tobacco at higher rates than the general population [1,11-13], which partially explains the increased risk of cardiovascular disease in this population. Bobes *et al*. [1] found that tobacco users with major psychotic or affective/mood disorders were more likely to consume daily alcohol and caffeine and less likely to avoid salt and saturated fats. Sedentary lifestyle and unhealthy food consumption patterns, including higher daily intake of calories and cholesterol, are common among individuals with major psychotic and/or affective disorders [11,14].

### Previous interventions tested

Many interventions intended to decrease risk factors for metabolic syndrome, both pharmacological and non-pharmacological, have been tested and described in the literature. Systematic reviews and meta-analyses of interventions to control risk factors for metabolic syndrome [15-23] indicate that both pharmacological and non-pharmacological (that is, behavioral or educational) interventions can be effective in decreasing metabolic risk. The studies described in these systematic reviews generally take place in mental health settings, which may exclude those individuals with mental illness who receive treatment primarily in the primary care setting. Only one study included in these reviews [24] features a primary care-based intervention. Previous weight loss interventions conducted in the primary care setting have demonstrated efficacy in many populations [25-29].

### Mental illness in primary care

Individuals with major psychotic or affective/mood disorders who are not psychiatrically hospitalized are generally treated for physical and sometimes mental health disorders in primary care settings in the U.S. due to a lack of integrated medical and behavioral health programs. Several studies in the U.S. and elsewhere have noted the prevalence of major psychotic or affective/mood disorders in the primary care setting. Serrano-Blanco *et al*. [30] conducted a study with over 3,800 primary care patients and found that 29.9% had a diagnosis of major depressive disorder. Das *et al*. [31] screened 1,157 primary care patients and found that approximately 10% met diagnostic criteria for bipolar disorder. Blount [32] reported that 80% of individuals with a mental health disorder will see their primary care physician in a given year, while only 50% will see a mental health provider. Roca *et al*. [33] reported on a similar study of more than 7,900 primary care patients and found that 29% of patients had been diagnosed with major depressive disorder. Fernandez *et al*. [34] conducted a cross-sectional study in primary care and found that mood disorders are the second leading cause of quality-adjusted life years in the primary care setting. The loss of quality of life and prevalence of psychiatric disorders in primary care demonstrates a need for primary-care based interventions to decrease chronic comorbid conditions.

### Objectives of review

The prevalence of psychiatric disorders in primary care settings and the association between chronic mental and physical illness necessitates an exploration of primary care-based interventions to address these comorbid conditions. This review focuses on non-pharmacological, education-based interventions to address metabolic syndrome risk factors in patients with major psychotic or affective/mood disorders who are treated in the primary care setting. The emphasis is on metabolic syndrome risk factors because this combination of risk factors can lead to chronic illnesses and early mortality in this population [35]. Education-based interventions are important because they empower the patient to manage his/her illness independently and expand the role of social workers in the primary care setting. According to Michie, Fixsen, Grimshaw and Eccles [36], systematic reviews of behavior change interventions typically produce modest effects. The primary author was involved in a primary care-based complex intervention to improve metabolic risk factors among patients with major psychotic and/or affective/mood disorders and patient reports indicated that they found the educational components most beneficial for self-management of illnesses [37].

## Methods

### Literature search

Electronic searches were conducted using MEDLINE, PsychINFO and the trials registry of the Cochrane Database of Systematic Reviews. The abstracts, titles and index terms of studies were searched in MEDLINE and PsychINFO using the following keywords: “schizophreni*” OR “schizoaffective” OR “bipolar” OR “major depressive disorder” OR “posttraumatic stress disorder” OR “serious mental illness” AND “metabolic syndrome” OR “high blood pressure” OR “triglycerides” OR “cholesterol” OR “HDL” OR “waist circumference” OR “blood sugar” OR “blood glucose” AND “intervention” OR “randomized controlled trial” OR “quasi-randomized” AND “primary care.” All titles in the Cochrane Schizophrenia Register were scanned for possible inclusion. Additionally, manual searches were conducted using references from literature found in the database search.

### Eligibility assessment

#### Inclusion and exclusion criteria

Studies were included if the population studied met the following criteria: adults ages 18 or older; diagnosed with one of the five mental illnesses which typically constitute major psychotic and/or affective disorders (schizophrenia (DSM-V code 295.9), schizoaffective disorder (DSM-V code 295.7), major depressive disorder (DSM-V code 296.3), or bipolar disorder (DSM-V codes 296.4 and 296.5)); and had risk factors for metabolic syndrome, including large waistline, a high triglyceride level, a low HDL cholesterol level, high blood pressure, and high fasting blood sugar level. The study setting must have been in a primary care location. The study design must have been either a randomized controlled trial (RCT) or a quasi-experimental study. Study outcomes must include one of the risk factors for metabolic syndrome (for example, blood pressure, waist circumference, triglyceride levels, blood glucose or (an increase in) HDL).

Studies were excluded if:

•the population studied was younger than 18 years old or did not have a diagnosis of a major psychotic and/or affective disorders or risk factors for metabolic syndrome;

•they were conducted in an inpatient or mental health-based setting;

•they were not an RCT or quasi-experimental study, and

•the outcome of the study did not include one of the risk factors for metabolic syndrome.

## Results

Our initial systematic search of databases MEDLINE and PsychINFO (which was not limited by setting) yielded 316 results. When "primary care" was added to the search, we found 19 additional results. A title search of the Cochrane Database of Systematic Reviews resulted in the identification of two systematic reviews; one review had no included studies and the other had six studies included in the quantitative synthesis. These studies were also identified in database searches and are part of the 363 total studies identified below. Manual searches from reference lists of articles found in the database search were conducted and 90 studies were found. A search of gray literature was conducted to decrease risk of publication bias using Open Grey ( http://www.opengrey.eu) with the same MESH terms, but no additional studies were found. A total of 363 unique studies were found from the collection of searches after duplicates were removed. The titles of all 363 of these results were reviewed separately by each reviewer and 303 were excluded based on setting or nature of intervention. There were no disagreements during this process. The remaining 60 articles were reviewed in abstract and 30 were excluded based on study design or setting. Full-text reviews were conducted by both reviewers CN and SJ for the remaining 30 of the studies and reviewers agreed that no studies met inclusion criteria; all studies were excluded. Additional file 1 provides a diagram of how studies were excluded. Table 1 lists all of the studies reviewed in full-text from database searches and manual searches with reasons for exclusion.

**Table 1:** Studies reviewed in full-text

**Authors, year**	**Reason for exclusion**
Alvarez-Jiminez *et al*., [38]	Partially pharmacological intervention, mix of different settings (including primary care).
Attux, Martini, de Araujo, Roma, Reis and Bressan, [39]	Not RCT; not primary care (mental health services)
Ball, Coons and Buchanan, [40]	Not primary care (both arms from outpatient MH services); not randomized
Bradshaw, Lovell and Harris, [41]	Not an RCT, not primary care
Brar *et al*., [42]	Not primary care
Brown, Goetz, Van Sciver, Sullivan and Hamera, [43]	Not primary care
Centorrino *et al*., [44]	Not primary care; no control group
Chafetz, White, Collins-Bride, Cooper and Nickens, [45]	Not primary care
Druss, Rohrbaugh, Levinson and Rosenheck, [24]	Wrong outcome
Evans, Newton and Higgins, [46]	Not primary care
Fosberg, Bjorkman, Sandman and Sandlund, [47]	Not primary care
Jean-Baptiste *et al*., [48]	Not primary care
Jones, Basson, Walker, Crawford and Kinon, [49]	Pharmacological intervention
Kalarchian *et al*., [50]	Not an RCT, not primary care
Khazaal *et al.,*[51]	Not primary care
Kilbourne *et al*., [52]	Not primary care; outcome not physical health
Kwon *et al*., [53]	Not primary care
Littrell, Hilligoss, Kirshner, Petty and Johnson, [54]	Not primary care, partially pharmacological
Mauri *et al*., [55]	Not primary care
McKibbin *et al*., [56]	Not primary care
Ohlson, Treasure and Pilowsky, [57]	Not RCT; not primary care
Park, Usher and Foster, [58]	Review paper
Pendlebury, Bushe, Wildgust and Holt, [59]	Not primary care, no control group
Perlman *et al*., [60]	Not RCT; not primary care
Poulin *et al*., [61]	Not primary care
Rotatori, Fox and Wicks, [62]	Not primary care
Skrinar, Huxley, Hutchinson, Menninger and Glew, [63]	Not primary care
Vreeland *et al.,*[64]	Not primary care, not randomized
Weber and Wyne, [65]	Not primary care
Weber and Nelson, [66]	Not RCT; not primary care

Through the manual search, 13 systematic reviews were identified for further review of citations. Table 2 provides a list of the 13 systematic reviews. These reviews examined a total of 221 studies. Raters CN and SJ independently screened titles or abstracts from these studies and all 221 studies were rejected for not meeting the inclusion criteria.

**Table 2:** Systematic reviews

**Authors, year**	**Title**	**# of studies reviewed in article**	**Conclusions**
Alvarez-Jiminez, Hetrick, Gonzalez-Blanch, Gleeson and McGorry, [15]	Non-pharmacological management of antipsychotic-induced weight gain: systematic review and meta-analysis of randomized controlled trials	10	Individual and group interventions, cognitive behavioral therapy and nutritional counseling were more effective than treatment as usual.
Bradshaw, Lovell and Harris, [16]	Healthy living interventions and schizophrenia: a systematic review	16	Inconclusive based on poor quality of studies reviewed.
Cabassa, Ezell and Lewis-Fernandez, [67]	Lifestyle interventions for adults with serious mental illness: a systematic literature review	23	Behavioral interventions generally showed improvement in metabolic syndrome risk factors
Caemmerer, Correll and Maayan, [68]	Acute and maintenance effects of non-pharmacological interventions for antipsychotic induced weight gain and metabolic abnormalities: a meta-analytic comparison of randomized controlled trials	18	Behavioral interventions effectively prevented and reduced weight gain in outpatients agreeing to participate in trials. Nutritional and cognitive behavioral interventions were effective.
Cimo,Stergiopoulis, Cheng, Bonato and Dewa, [69]	Effective lifestyle interventions to improve type 2 diabetes self-management	4	Diabetes education is effective when it includes diet and exercise and design should address cognition, motivation and weight gain
Faulkner, Soundy and Lloyd, [17]	Schizophrenia and weight management: a systematic review of interventions to control weight	16	All behavioral interventions produced small reductions in, or maintenance of, weight.
Gabriele, Dubert and Reeves, [18]	Efficacy of behavioural interventions in managing atypical antipsychotic weight gain	16	When behavioral interventions were initiated at the start of atypical antipsychotic (AAP) treatment, amount of weight gain was decreased. When initiated after the start of AAP treatment, weight loss was achieved. Insulin regulation and A1c (metabolic syndrome risk factors) were also improved.
Megna, Schwartz, Siddiqui and Rojas, [19]	Obesity in adults with serious and persistent mental illness: a review of postulated mechanisms and current interventions	71	Non-pharmacological interventions are promising, but only show low to medium effect size.
Papanastasiou, [20]	Interventions for the metabolic syndrome in schizophrenia: a review	15	Behavioral interventions showed benefit, but study design (non-RCT) did not prove one intervention superior to another.
Roberts and Bailey, [21]	Incentives and barriers to lifestyle interventions for people with severe mental illness: a narrative synthesis of quantitative, qualitative and mixed methods studies	14	No studies identified that specifically focus on incentives and barriers
Tosh. Clifton, Mala and Bachner, [70]	Physical health care monitoring for people with serious mental illness	0	No studies identified that specifically focus on incentives and barriers.
Tosh, Clifton and Bachner, [22]	General physical health advice for people with serious mental illness	6	Health advice could lead to greater access of services but ineffective advice may be a waste of resources.
Werneke, Taylor, Sanders and Wessely, [23]	Behavioral management of antipsychotic-induced weight gain: a review	12	No RCTs identified, but interventions appear to be effective.
	Total	221	

## Discussion

Although we identified no studies that met the *a priori* inclusion criteria, there were 16 studies identified during database and manual searches that examined similar interventions in non-primary care settings. These studies (listed in Table 3) demonstrate that controlled trials with education interventions to improve physical health can be conducted with individuals with major psychotic and/or affective disorders; the systematic reviews shown in Table 2 indicate that these interventions can be effective. Also, it should be recognized that no evidence of effective primary care-based studies does not mean that such intervention is ineffective; further studies are needed in this area to determine whether such interventions can be effective in primary care settings.

**Table 3:** Similar interventions not in primary care settings

**Authors, year**	**Title**	**Setting**	**Description of intervention**	**Length of intervention**	**Appropriate for primary care**
Brar *et al*., [42]	Effects of behavioral therapy on weight loss in overweight and obese patients with schizophrenia or schizoaffective disorder	Mental health	Manual-based behavioral techniques for weight loss	14 weeks	Yes
Brown, Goetz, Van Sciver, Sullivan and Hamera, [43]	A psychiatric rehabilitation approach to weight loss	Mental health	Goal setting, social support, skills training, more frequent visits with providers, meal replacements	12 weeks	No
Chafetz, White, Collins-Bride, Cooper and Nickens, [45]	Clinical trial of wellness training: health promotion for severely mentally ill adults	Short term residential treatment	Promoting individual skills in self-management of illness	12 months	No
Evans, Newton and Higgins, [46]	Nutritional intervention to prevent weight gain in patients commenced on olanzapine: a randomized controlled trial	Mental health	Nutrition education sessions	12 weeks	No
Fosberg, Bjorkman, Sandman and Sandlund, [47]	Physical health – a cluster randomized controlled lifestyle intervention among persons with a psychiatric disability and their staff	Residential mental health	Curriculum including motivation, food content, stress and fitness	12 months	No
Jean-Baptiste *et al*., [48]	A pilot study of a weight management program with food provision in schizophrenia	Mental health	Weekly group sessions w/dietitian and psychiatrist, pedometers and food (or reimbursement) provided, individual nutrition support, grocery store visit	16 weeks	No
Khazaal *et al*., [51]	Cognitive behavioral therapy for weight gain associated with antipsychotic drugs	Mental health	Cognitive behavioral therapy	12 weeks	Yes
Kilbourne *et al*., [52]	Improving medical and psychiatric outcomes among individuals with bipolar disorder: a randomized controlled trial	Mental health	Self-management sessions on bipolar disorder, promotion of provider engagement, education related to cardiovascular disease	4 weeks	Yes
Kwon *et al*., [53]	Weight management program for treatment-emergent weight gain in olanzapine-treated patients with schizophrenia or schizoaffective disorder: a 12-week randomized controlled trial	Mental health	Educational program with food diary, nutrition education, exercise management	12 weeks	Yes
Mauri *et al.,*[55]	A psychoeducational program for weight loss in patients who have experienced weight gain during antipsychotic treatment with olanzapine	Mental health	Weekly psycho-educational meetings emphasizing weight loss with personalized diet plans	24 weeks	No
McKibbin *et al*., [56]	A lifestyle intervention for older schizophrenia patients with diabetes mellitus: a randomized controlled trial	Residential mental health	Diabetes Awareness and Rehabilitation Training (DART)	24 weeks	Yes
Mcreadie *et al*., [71]	Dietary improvement in ppl with schizophrenia: randomized controlled trial	Residential mental health	Giving fruit, veggies and meal planning to patients (vs. fruit/vegetables alone)	6 months	No
Poulin *et al*., 2007 [61]	Management of antipsychotic induced weight gain: prospective naturalistic study of the effectiveness of a supervised exercise programme	Mental health	Education, physical education counseling and exercise	18 months	No
Rotatori, Fox and Wicks, [62]	Weight loss with psychiatric residents in a behavioral self-control program	Inpatient mental health	Behavior therapy	14 weeks	No
Skrinar, Huxley, Hutchinson, Menninger and Glew, [63]	The role of a fitness intervention on people with serious psychiatric disabilities	Mental health	Exercise, weekly education seminars	12 weeks	Yes
Weber and Wyne, [65]	A cognitive behavioral group intervention for weight loss in patients treated with atypical antipsychotics	Mental health	Based on Diabetes Prevention Project (DPP) program to prevent diabetes	16 weeks	Yes

Of the 16 similar studies of educational interventions, reviewers identified 6 studies that may be able to be implemented in the primary care setting [42,51,53,56,63,65]. Those studies of interventions that might not be appropriate for primary care include interventions that were too long [45,47,61], provided products or services that might not be available in primary care settings [43,48,55,62,71] or required patients to have not yet developed physical risk factors prior to the intervention [46].

An examination and discussion of the details of the interventional components of the studies possible in primary care identified in Table 3 is warranted here, because future studies in the primary care setting must adequately describe their interventions in order to be replicated or subject to systematic review. The methodological quality of these studies is summarized in Table 4. All of these studies provided explicit descriptions of the intervention components; McKibbin *et al*. [56], Weber and Wyne [65] and Kwon *et al*. [53] also described session-by-session content of the intervention in table and narrative format; Brar *et al*. [42] described sessions in narrative format only. Khazaal *et al*. [51] used an intervention previously developed by one of the authors, so readers can review that intervention in detail elsewhere, but it was not described in detail in the article. Srkinar *et al*. [63] provided a description of the length of the educational intervention and a list of topics, but no sequence or table of sessions was provided. All studies identified as possible in primary care included an intervention element that was not education (or example, food tasting, exercise sessions, provision of pedometers), so the effectiveness of the educational component alone may not be able to be determined from these studies; however, complex interventions are very common in behavioral health research [72].

**Table 4:** Methodological quality of studies appropriate for primary care

**Author, date**	**Type of study**	**Sample size**	**Power analysis described**	**How participant characteristics described**	**Review board approved**	**Randomization process described**	**Comparator**	**Blinding described**	**Evidence base for intervention described**	**Statistical analysis described**
Brar *et al*., [42]	RCT	71	Yes	Narrative	Yes	No	Monthly weight checks and encouragement of weight loss	No	Yes; table with previous studies	Yes (paired *t*-tests, logistic regression, ANCOVA, Cochran-mantel-Haenszel)
Khazaal *et al*., [51]	RCT	61	No	Table	Yes	No	Control group with brief nutritional education	No	Yes; previous "Apple Pie" study	Yes (*t*-tests, Chi-square, MANOVA, MANCOVA, Fisher's exact significance tests, Cochran's Q test)
Kwon *et al*., [53]	RCT	48	Yes	Table	Yes	No	Routine care with verbal diet and weight management recommendations. Control group also given food and exercise diaries.	No	No	Yes (*t*-test and ANCOVA)
McKibbin *et al*., [56]	RCT	64	No	Table	Yes	No	Usual care with three health-related brochures distributed	No	Yes; previous DART study and theoretical orientation	Yes (ANOVA, t-tests, chi-square)
Skrinar, Huxley, Hutchison, Menninger and Glew, [63]	RCT	20	No	Table	Yes	No	Waiting list control group	No	Yes; cites literature about role of exercise in weight management	Yes (ANOVA)
Weber and Wyne, [65]	RCT	17	No (pilot study)	Table	Yes	No	Control group received treatment as usual and were weighed every four weeks.	Yes	Yes; previous Diabetes Prevention Project	Yes (*t*-test*t*-test, statistical significance set at 0.05)

Missing from the descriptions of many articles reviewed in this study were details about who implemented an intervention and where it took place. A number of studies stated that participants were recruited from a certain hospital or facility (for example, Khazaal *et al*. [51] and Skrinar *et al*. [63], but it was not clear from the articles whether the actual educational intervention took place in the hospital or in an outpatient setting. Of the studies identified as possible in primary care, only Khazaal *et al*. [51], Kwon *et al*. [53] and Weber and Wyne [65] provided a clear description of who was implementing the intervention, so it is not clear if academic researchers, dietitians, medical professionals or social workers were implementing the other interventions. It is also not clear in several studies, including Srkinar *et al*. [63] and Khazaal *et al*. [51], who was collecting any of the data, which could affect participant outcomes (for example, if the patients had an existing relationship with the data collectors) and may be subject to detection bias if assessors were not blind to allocation.

Health outcomes from complex behavioral interventions can be nebulous because multiple factors affect outcomes; however, the RCT format of the studies discussed here improves study rigor [73]. Two studies [63,65] resulted in no statistically significant reduction in metabolic risk factors, with both studies citing small sample sizes and other factors (for example, lack of transportation, motivation) as being possible explanations for these results. Khazaal *et al*. [51] found limited reduction in weight and body mass index (BMI) in the experimental group. Some subjects’ medications were also changed during the study, although the authors used statistical methods to account for the possible impact of these changes [51]. McKibbin *et al*. [56] and Kwon *et al*. [53] reported significant reductions in metabolic syndrome risk factors (weight, BMI) as a result of their interventions. Kwon *et al*. [53] also observed significant weight loss in the control group, which suggests possible threats to internal validity in the design. These authors do note that several members of the experimental group lost a greater percentage of body weight than anyone in the control group.

## Conclusions

The authors were unable to identify rigorous, primary care-based interventions to address physical illness among individuals with mental illness. As the literature expands to include primary care-based interventions, additional systematic reviews and meta-analyses are warranted to assess effectiveness in this setting. Systematic reviews of high-quality RCTs are the most rigorous form of effectiveness research, as single RCTs can have weak designs or biased results [73]. Quality assurance protocols, such as the CLEAR NPT checklist for non-pharmacological trials [74], which provides a checklist for components of quality in a study, should be incorporated into future studies in this area to provide standardized guidelines for making effectiveness claims.

Adequate reporting of interventional content and components is also essential to the expansion of literature in this subject area and groups, such as the Workgroup for Intervention Development and Evaluation Research (WIDER), have developed suggestions for intervention reporting ( http://interventiondesign.co.uk). WIDER advocates for the successful adoption of behavior change interventions and the expansion of CONSORT ( http://www.consort-statement.org) and APA guidelines to allow for improved reporting of these interventions. In behavioral intervention research, theories regarding the specific mechanism of change within an intervention should be utilized during the development of the intervention and should be described in the final report [36].

Social work researchers and direct service social workers in health care settings have an opportunity to design and implement high-quality behavioral and educational programs for individuals with major psychotic and/or affective disorders using the criteria described above. Social workers are among the few professionals in health care settings who have the skills and opportunity to work closely with the patients most in need of health-related behavior change interventions, as they are generally able to spend more time with patients in health care settings and have access to medical information in collocated health and mental health settings. Interventions to decrease metabolic syndrome risk factors have been demonstrated to be successful in mental health settings, but the primary service in mental health settings is mental health. Primary care-based interventions are important for conveying the message that the focus is on physical health, even if the population is comprised of individuals with major psychotic and/or affective disorders. Social workers or social work researchers participating in health-focused interventions that do not follow published guidelines for research and reporting of RCTs are missing an important opportunity to enhance systematic reviews of literature about this population.

## Abbreviations

AAP: Atypical antipsychotic medication; BMI: Body mass index; DART: Diabetes Awareness and Rehabilitation Training; RCT: Randomized control trial

## Competing interests

The authors declare that they have no competing interests.

## Authors’ contributions

CN conceived of the study, developed the search terms, conducted the literature search, reviewed studies and drafted the manuscript. SJ served as the second reviewer, developed the tables and assisted with drafting the manuscript. CN and SJ together edited the manuscript. Both authors read and approved the final manuscript.

## Supplementary Material

Additional file 1 **PRISMA diagram for BMC.** PRISMA 2009 Flow Diagram. PRISMA diagram of studies excluded. Click here for file
