# Supplementary material for: Primary care-based educational interventions to decrease risk factors for metabolic syndrome for adults with major psychotic and/or affective disorders: a systematic review
Source: Syst Rev. 2013 Dec 27;2:116. doi: 10.1186/2046-4053-2-116 (PMC3877871; doi:10.1186/2046-4053-2-116)
Supplement: Additional file 1 — PRISMA diagram for BMC. PRISMA 2009 Flow Diagram. PRISMA diagram of studies excluded. [file 2046-4053-2-116-S1.doc]

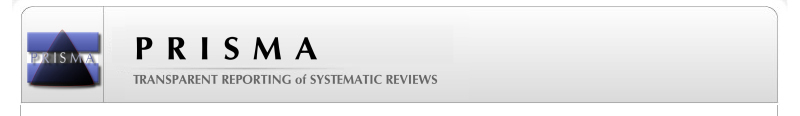
**PRISMA 2009 Flow Diagram**

**Screening**

**Included**

**Eligibility**

**Identification**

Records identified through database searching
(n = 337 )

Additional records identified through other sources
(n = 90 )

Records after duplicates removed
(n = 361 )

Records screened
(n = 421)

Records excluded
(n =391)

Full-text articles assessed for eligibility
(n = 30 )

Full-text articles excluded, with reasons
(n =30 )

Studies included in qualitative synthesis
(n = 0 )

Studies included in quantitative synthesis (meta-analysis)
(n = 0 )
